# Climate shocks and wealth inequality in the UK: evidence from monthly data

**DOI:** 10.1007/s11356-023-27342-1

**Published:** 2023-06-01

**Authors:** Xin Sheng, Carolyn Chisadza, Rangan Gupta, Christian Pierdzioch

**Affiliations:** 1 Lord Ashcroft International Business School, Anglia Ruskin University, Chelmsford, UK; 2 Department of Economics, University of Pretoria, Private Bag X20, Hatfield, 0028 South Africa; 3 Department of Economics, Helmut Schmidt University, Holstenhofweg 85, P.O.B. 700822, 22008 Hamburg, Germany

**Keywords:** Climate change, Climate shock, Wealth inequality, UK, Temperatures, D63, O13, O51

## Abstract

This paper investigates both the linear and nonlinear effects of climate risk shocks on wealth inequality in the UK using the local projections (LPs) method, based on high-frequency, i.e., monthly data. The linear results show that climate risk shocks lead to an increase in wealth inequality in the longer term. The nonlinear results present some evidence of heterogeneous responses of wealth inequality to climate risk variable shocks between high- and low-climate risk regimes. The findings highlight the disproportionate increased burden of climate change on households that are already experiencing poverty, particularly households in high-climate risk areas. As such, measures to mitigate the adverse effects of climate change need to be tailored so as not to overburden the poor.

## Introduction

Reducing wealth inequality between countries continues to be a challenging global agenda, which threatens to derail sustainable development. In recent decades, progress towards reducing wealth inequality between countries has been hindered by climate change, with an estimated 68 to 135 million people being pushed into poverty by 2030 because of climate change (Guivarch et al. 2021). In addition, in the past year of 2021, the world has experienced high temperatures in the Pacific Northwest that killed over 200 people, severe flooding in Western Europe and parts of Africa, as well as drought and heatwaves in Central Asia.^1^ These are just a few of the alarming examples that highlight the serious need to find solutions to managing the adverse effects of climate change.

While there is much discussion of how climate change affects inequality between countries in the literature (Roberts 2001; Hsiang & Jina 2014; Taconet et al. 2020), less attention is drawn to the climate change risk on economic inequality within countries.^2^ When climate change reduces productivity, the economic situation of vulnerable groups in society, such as low-income households, is made more insecure. For example, recent evidence highlights that climate change can widen wealth inequality between income groups within countries through increased risks to investments, food security or education attainment (Colmer 2021; Park et al. 2020). According to Colmer (2021), higher temperatures can affect people’s investments if climate-induced scarcity reduces savings at low-income levels. In addition, Park et al. (2020) report evidence that higher temperatures affect children’s learning outcomes in poorer school districts more than in rich districts, thus increasing differences in educational attainment. Given that vulnerable groups in society already struggle with social and health inequalities (e.g. access to credit, access to healthcare services, or access to quality education), these inequalities can be further reinforced by climate-induced wealth inequality, increasing poor people’s vulnerability, reducing their capacity to adapt to changing environment and causing them to be caught in a poverty trap. To mitigate these potentially adverse distributional effects, developing a better understanding of the climate change-inequality nexus is imperative.

The dearth of empirical literature on climate change and wealth inequality within countries leaves scope for more evidence-based studies to unpack the mechanisms that can explain the adverse effects of climate change on wealth inequality. Understanding these effects is key if we are to formulate effective climate change strategies that mitigate the damage while improving the resilience of people at all income levels. In this regard, we contribute to the literature by examining the relationship between climate risk shocks and wealth inequality in the UK. We find that rising temperatures increase wealth inequality in the long run. We also find evidence of heterogeneous responses of wealth inequality to climate risk shocks between high- and low-climate risk regimes.

The findings contribute to the climate change discussion by firstly providing a shift in the existing narrative that is typically weighted relatively more towards climate risks disproportionately affecting poorer countries (Intergovernmental Panel on Climate Change 2012; Ashenafi 2022). Evidence is emerging that rich countries, which tend to be underexplored in the climate change literature, are not as immune to the impact of climate change as poor countries (Knight et al. 2017). Even within rich countries, poorer people can also be more vulnerable to the impact of climate change. For example, the UK recorded the highest number of heatwave deaths in 2020.^3^ At the same time, inequality has been on the rise in the UK (Brewer & Wren-Lewis 2016; Mumtaz & Theophilopoulou 2017).

The UK has among the highest level of income inequality in relation to other developed countries in Europe (Dorling 2015), with the richest fifth earning an income twelve times the amount that is earned by the poorest fifth (Office for National Statistics 2019). Moreover, in terms of wealth, the richest 10% of households hold about 44% of all the wealth, while the poorest 50% hold only 9% (Office for National Statistics 2018). However, besides the standard factors of growth, inflation, monetary and fiscal policies, and while several other macroeconomic and financial drivers of increasing inequality in the UK have been discussed in the literature, such as the term spread, globalisation, income volatility, household debt, labour productivity, and even financial stress (Aye et al. 2020; Mumtaz & Theophilopoulou 2020; Berisha et al. 2021, forthcoming; Gabauer et al. 2021; Balcilar et al. 2022; Pierdzioch et al. 2022), there is limited empirical evidence on the effects of climate risk in the UK, particularly on wealth distribution. Regardless of a country’s level of development or income ranking, climate change threatens to reverse development gains if unabated, especially given the fact that physical or transition risks^4^ associated with climate change are likely to affect all future realizations of macroeconomic and financial variables (Giglio et al. 2021; Sheng et al. 2022a; 2022b), which can be potentially associated with movements in wealth inequality.

Secondly, unlike existing empirical evidence that relies on annual (low-frequency) data, we compute impulse response functions from the local projections method using high-frequency (monthly) data. High-frequency data allows for better flexibility in modelling the associations between outcome and treatment variables of interest by eliminating noises from other factors. Using high-frequency data captures more information on the shocks that the UK may have been subject to over the available sample period, thus making the predictions of the model more accurate when detecting the reactions of inequality to climate shocks than when using low-frequency data (Boudt et al. 2015; Mumtaz & Theophilopoulou 2020). As such, high-frequency analysis of climate risks on inequality assists in gaining insightful knowledge about the climate change-inequality dynamics and supports the better design of policymaking. For example, identifying the adverse impacts across income groups in the immediate period and implementing early interventions for those income groups most affected by climate risk to avoid prolonging the negative effects and potentially increasing poverty and wealth disparities in the long run.

### Related literature

Previous literature has offered some important insights into the linkages between climate change and economic development and the associated socio-economic costs (Gomez-Echeverri 2018; Donadelli et al. 2017). Various mechanisms have been identified through which climate change can have aggravating effects on sustainable development. According to Intergovernmental Panel on Intergovernmental Panel on Climate Change (2014), climate change negatively affects key development sectors, such as agriculture, health and education, as well as well-being through food security, access to water, conflict, poverty and inequality. These adverse impacts damage capital stock and labour productivity, which weakens economic growth (Colmer 2021; Donadelli et al. 2017). More than that, the impact of climate change on different sectors can have spill-over effects. For example, droughts can contribute to reduced crop yields in the agriculture sector, which threatens food security (Hallegatte & Rozenberg 2017; Wiebe et al. 2015; Müller et al. 2015), or can impact access to clean drinking water, which compromises human health. Flooding can lead to damage to ecosystems and infrastructure, while rising temperatures can create thriving environments for disease-carrying insects such as mosquitoes or fleas, which can also compromise human and domestic animal health leading to lower labour productivity (Burke et al. 2015; Hsiang et al. 2017). A study by OECD (2015) made quantitative assessments to the year 2060 and concluded that the projected negative effects of climate change will be greatest for agriculture and health sectors, with the worst damage in developing regions, such as Asia and Africa.

Another strand of climate change evidence is based on mitigation actions. Evidence by Rafaj et al. (2013) and West et al. (2013) shows how stringent climate mitigation strategies could improve air quality and lead to better life expectancy in Europe, China and India. Furthermore, a study by Markandya et al. (2015) in Uganda estimated that the damage to economic sectors could total 2 to 4% of the gross domestic product during the period 2010 to 2050, and that although the climate change adaptation costs would be high, the costs of inaction would be even higher by about 20 to 40 times. For instance, the International Organisation of Migration estimates that in 2008, more people were displaced by extreme weather events (about 20 million) than by conflict (about 4 million) (Flavell & Chazalnoël, 2014). These statistics are supported by findings from Castells-Quintana (2022) where exposure to floods is associated with higher intensity of urban conflict through the displacement of populations into larger cities. In the USA, extreme heat is a cause of high death rates relative to other natural disasters, such as tornadoes, hurricanes and lightning storms (Denchak 2022).

There is also growing evidence that climate change has increased global economic inequality, as well as within-country inequalities. A framework developed by Islam and Winkel (2017) identifies three pathways through which climate change can affect wealth inequality within countries. The first pathway emphasises that increased within-country inequality can occur due to an increase in exposure to climate change of the low-income groups based on their location. Evidence linked to this pathway suggests that lower-income neighbourhoods and communities are disproportionately exposed to environmental hazards (Mohai et al. 2009). Moreover, climate change affects people living in warmer regions where any additional increases in temperatures would have negative impacts on society (Diffenbaugh & Burke 2019). The second pathway highlights that an increase in vulnerability to damage caused by climate change is relatively worse for low-income groups than high-income groups due to a lack of resources or social protection. Evidence suggests that poorer countries or individuals are more negatively affected because they lack the resources to respond to climate change risks (Taconet et al. 2020). In addition, Ashenafi (2022) finds that greenhouse gas emissions widen inequality in poorer regions, such as Africa.

The third pathway indicates that a decrease in the low-income groups’ ability to cope and recover from climate change exacerbates existing wealth inequalities. For example, climate change can bring uncertainty which can affect how people respond to its effects, such as how much effort or resources should they be expending to mitigate the negative effects. Evidence in experiments by Brown and Kroll (2017) indicates that uncertainty lowers contributions toward reducing a threat, and this can be worsened if agents have income differences. According to Burton-Chellew et al. (2013), cooperation collapses when inequality in resources is combined with a greater relative risk for the poor. They argue that the rich invest proportionally less into preventing climate change when they are less at risk. This argument is collaborated by Knight et al. (2017) where research from the USA indicates that the rich are less supportive of environmental protection.

Moreover, Taconet et al. (2020) find that climate change is a main cause of inequality as it can delay the development convergence between poor and rich countries. Unfortunately, evidence linking climate change to inequality shows that human influences are among the top contributors to global warming through the burning of fossil fuels, which causes air pollution, and deforestation, which prevents the capture of air pollution. For example, wealthy countries have historically contributed to greenhouse gas emissions following the Industrial Revolution (Hartmann 2013). According to Guivarch et al. (2021), the richest countries make up about 16% of the world’s population and yet account for almost 40% of carbon dioxide emissions, while the poorer countries that make up 60% of the global population only account for 15% of the emissions.

The evidence cited here stresses the vital contribution that studies, such as this one, makes to understanding the dynamics of climate change and how it can affect sustainable development.

## Data and methodology

The wealth inequality measures are calculated based on the Wealth and Assets Survey (WAS) conducted by the Office for National Statistics (ONS) in the UK. The WAS samples private households in the UK and collects data about the values of total household wealth, including net property wealth, net financial wealth, private pension and physical wealth.^5^ The WAS allows for measures of changes in total wealth in UK households over time, and it is the only data source that allows for the construction of UK wealth inequality measures at a monthly frequency. We follow the work of Mumtaz and Theophilopoulou (2020) for the measures of wealth inequality in the UK. The wealth inequality measures of Mumtaz and Theophilopoulou (2020) are percentile ratios that compare the average wealth for households that locate in the left, middle or right tail of the total wealth distribution. The 80–20 ratio is defined as $\frac{{\overline{P} }_{80}}{{\overline{P} }_{20}}$, where ${\overline{P} }_{80}$ and ${\overline{P} }_{20}$ represent the average wealth for households that lie between the 75th and 85th percentile of total wealth and that lie between the 15th and 25th percentile of total wealth, respectively. The 90–10 ratio is defined as $\frac{{\overline{P} }_{90}}{{\overline{P} }_{10}}$, where ${\overline{P} }_{90}$ and ${\overline{P} }_{10}$ represent average wealth for households that lie between the 85th and 95th percentile of total wealth and that lie between the 5th and 15th percentile of total wealth, respectively (Mumtaz and Theophilopoulou 2020). The 80–20 (90–10) ratio compares the wealth of households around the top 20% (10%) of the distribution in the right tail to the wealth of households near the bottom 20% (10%) of the distribution in the left tail. Similarly, the 80–50 (90–50) and 50–20 (50–10) wealth inequality ratios capture how the wealthier households in the top 20% (10%) percentile and the poorer households in the bottom 20% (10%) percentile move relative to the households near the median of the total wealth distribution (Mumtaz and Theophilopoulou 2020).

The data for the UK monthly average temperature (in degrees Celsius) is collected from the Met Office in the UK. We use the year-on-year temperature growth and its volatility shocks as measures of climate risks. To this end, we calculate the residuals from the AR (12) model of climate risk variables (i.e., growth of temperature and its volatility) for the temperature growth shock and temperature growth volatility shock, respectively. Our sample period starts in July 2006 and ends in March 2018. Our data is available at a monthly frequency.

**Fig. 1:**
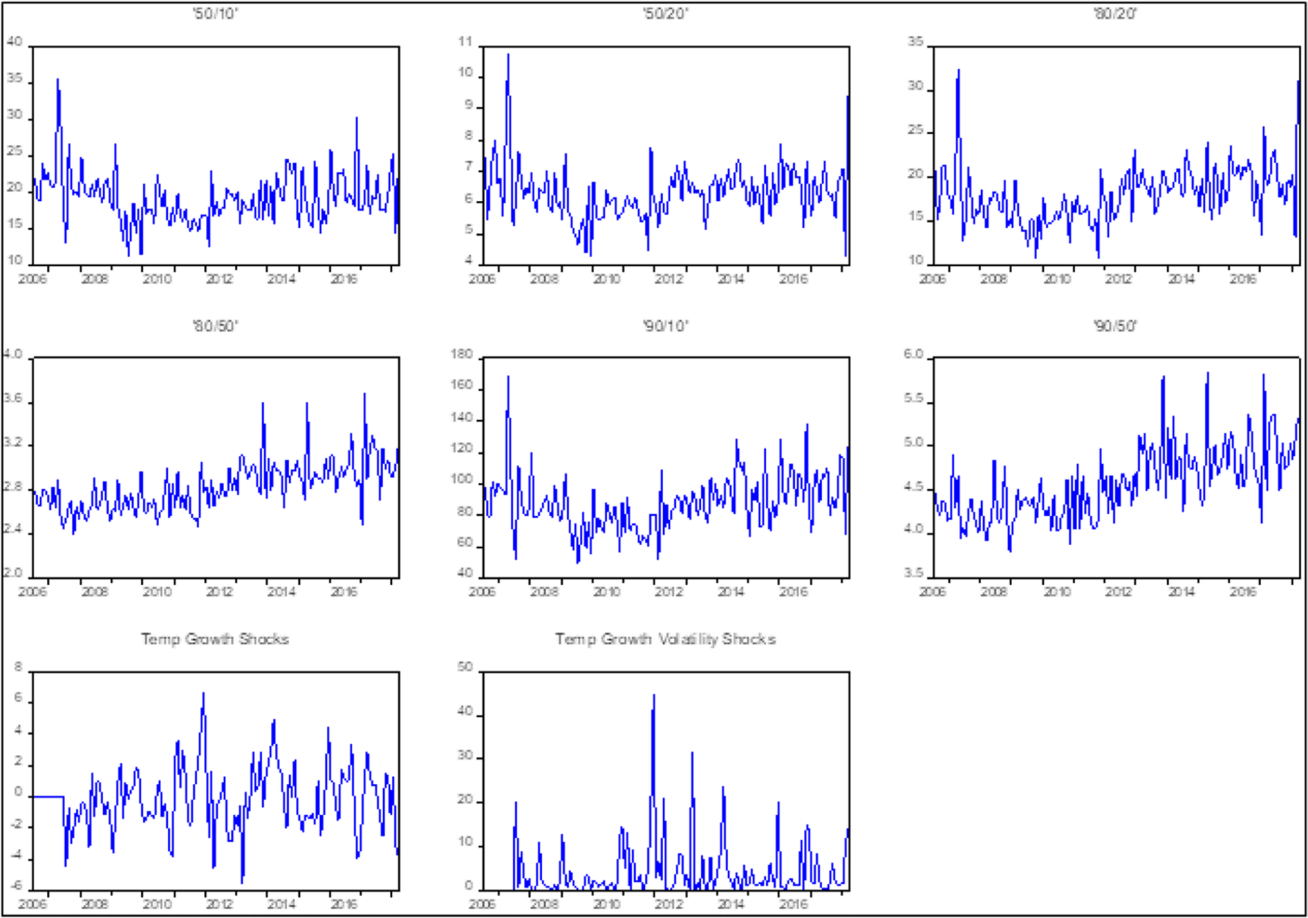
Data plots

In Table 1, we present basic descriptive statistics on various percentile-based wealth inequality measures as developed by Mumtaz and Theophilopoulou (2020) and the UK monthly average temperature over the sample period from July 2006 to March 2018. The statistics show that the mean values of wealth inequality ratios range from 2.83 (for the 80–50 ratio) to 87.87 (for the 90–10 ratio). The high mean value of the 90–10 measure indicates a large wealth inequality between the average wealth of households around the top 10% of the distribution and the average wealth of households near the bottom 10%. In contrast, the 80–50 ratio, which measures how the wealthier households in the top 20% percentile move relative to the households near the median of the total wealth distribution, has the smallest mean value. Among the various wealth inequality ratios, the maximum value of 168.56 is observed for the 90–10 ratio, while the minimum value of 2.40 is observed for the 80–50 ratio. For the UK monthly average temperature, the mean value over the sample period is 10.14° (in Celsius), while the highest monthly average temperature is recorded as 19.70° and the lowest one is − 0.70°. In Fig. 1, we also display data plots on various percentile-based wealth inequality measures and the temperature growth and temperature growth volatility shocks over the sample period. The dynamics of various measures of wealth inequality can be seen in data plots in the first two rows of Fig. 1. It is useful to note that, the onset of the 2007–2008 global financial crisis (GFC) coincided with a short-lived but sharp increase in the wealth inequality measures for the 50–10, 50–20, 80–20, and 90–10 ratios. However, no such spike was observed for the 90–50 and 80–50 measures.

**Table 1:** Descriptive statistics

	’50–10’	’50–20’	’80–20’	’80–50’	’90–10’	’90–50’	Temp
Mean	19.21	6.32	17.99	2.83	87.87	4.57	10.14
Median	18.80	6.29	17.97	2.81	86.71	4.51	10.20
Maximum	35.48	10.74	32.38	3.68	168.56	5.84	19.70
Minimum	11.19	4.28	10.84	2.40	48.76	3.79	− 0.70
Std. Dev	3.54	0.88	3.28	0.23	18.20	0.41	4.61
Observations	141	141	141	141	141	141	141

We estimate linear impulse response functions (IRFs) by means of the local projections (LPs) technique pioneered by Jordà (2005).^6^ Accordingly, the linear model is specified as follows:

1 $${WI}_{t+s}={\alpha }_{s}+{{\beta }_{s}CR}_{t}+{\epsilon }_{t+s}, \mathrm{for }s=\mathrm{0,1},2, \dots H$$

where ${WI}_{t}$ represents the log level of wealth inequality ratios in the UK at time *t,* and *s* is the length of forecast horizons up to the maximum forecast horizon *H.* We set *H* = 12, that is, the maximum forecast horizon is 12 months (1 year). The parameters ${\beta }_{s}$ capture the response of wealth inequality at time *t* + *s* to a shock to climate risk variables (denoted by ${CR}_{t}$) at time *t*. The lags of the AR models are determined by the AIC/BIC criteria. We then compute the IRFs from a series of ${\beta }_{s}$ that are estimated separately by the ordinary least squares (OLS) regression technique at each horizon (*s*).^7^

To estimate the impacts of climate shocks on UK wealth inequality, we also control for a large set of macroeconomic and financial variables following the work of Mumtaz and Theophilopoulou (2020). The model specified in Eq. (1) can be further extended by adding the principal components of 38 economic and financial time series as the control variable to account for a large information set.^8^ The model can be re-specified as follows.

2 $${WI}_{t+s}={\alpha }_{s}+{{\beta }_{s}CR}_{t}+{{\gamma }_{s}CV}_{t}+{\epsilon }_{t+s}, \mathrm{for }s=\mathrm{0,1},2, \dots H$$

where ${CV}_{t}$ represents the control variable at the monthly frequency.

Using the loca projection approch, we also study whether the effect of climate risks on wealth inequality is regime-dependent in the sense that the effects depend on the high and low regimes of the climate risk variables.^9^

3 $${WI}_{t+s}=\left(1-F\left({z}_{t-1}\right)\right)\left[{\alpha }_{s}^{High}+{\beta }_{s}^{High}{CR}_{t}\right]+F\left({z}_{t-1}\right)\left[{\alpha }_{s}^{Low}+{\beta }_{s}^{Low}{CR}_{t}\right]+{\gamma }_{s}{CV}_{t}+{\epsilon }_{ t+s}, \mathrm{for }s=\mathrm{0,1},2, \dots H$$

4 $$F\left({z}_{t}\right)=exp\left(-\gamma {z}_{t}\right)/1+exp\left(-\gamma {z}_{t}\right),\gamma >0$$

where ${z}_{t}$ is a switching variable measuring the high and low regimes of the climate risk variables. We normalize ${z}_{t}$ so that it has zero mean and unit variance, with a positive value of ${z}_{i,t}$ indicating high regimes of the climate risk variables, and a negative value otherwise. The smooth transition function $F\left({z}_{t}\right)$ is bounded between 0 and 1, with values close to 1 corresponding to low regimes of the climate risk variables, and 0 otherwise.

## Results

**Fig. 2:**
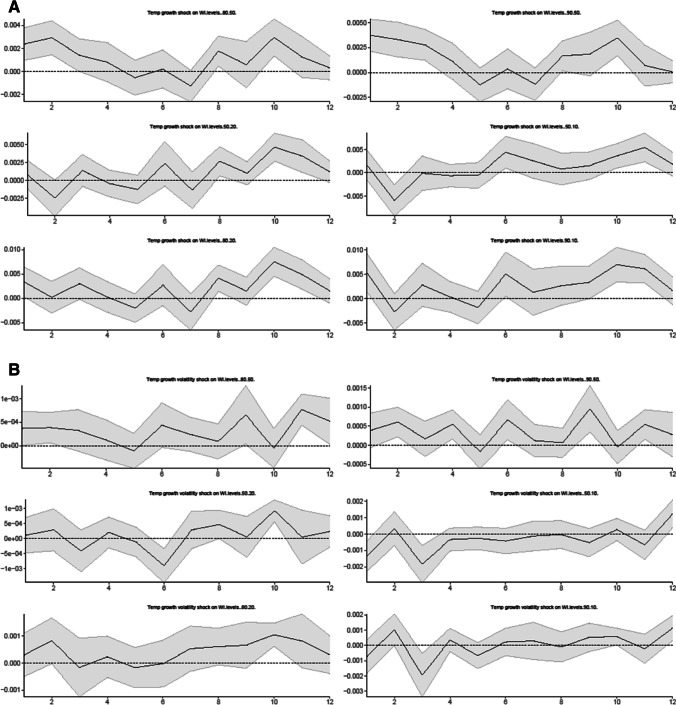
Linear responses of wealth inequality to a climate risk shock (without the control variable). **A** Temperature growth shock on wealth inequality, **B** Temperature growth volatility shock on wealth inequality

In Fig. 2, the linear IRFs results depicted in Fig. 2A and B show how various wealth inequality measures (i.e., the 80–50, 50–20, 80–20, 90–50, 50–10, and 90–10 ratios) react to climate risk shocks (i.e., the temperature growth shock and its volatility shock) over the 12-month forecast horizon.

Our results show that both temperature growth and volatility shocks have positive and statistically significant effects on all measures of wealth inequality in the longer term, (e.g., temperature growth shock on all wealth inequality measures in the 10th month as reported in Fig. 2A, and temperature volatility shock on various wealth inequality measures in the 10^th^, 11^th^ or 12^th^ month as reported in Fig. 2B).^10^ This result is in line with recent literature on climate hazards and economic inequality nexus. For example, Paglialunga et al. (2020, 2022) investigate the impact of various measures of climate variability on income inequality for more than 150 nations and find that the temperature anomaly can be a key driver of within-country inequality. Paglialunga et al. (2020, 2022) report strong empirical evidence that temperature increases have a statistically significant effect on driving up inequality. Burzyński et al. (2022) also find that climate change exerts a strong influence on the distribution of income and wealth globally, and it deepens inequality.

Our results also show that a shock to climate risk variables increases wealth inequality as measured by the 80–20 (90–10) ratio by a larger amount than other wealth inequality ratios such as the 80–50 (90–50) and 50–20 (50–10) measures, indicating that climate risk shocks harm the poorest the most relative to the richest households, exacerbating wealth inequality in a longer term. According to Simms et al. (2009, https://neweconomics.org/2009/01/tackling-climate-change-reducing-poverty), the most likely to suffer if climate change continues unabated will be the low income households in the UK because (a) they live in cheaper and lower quality housing with poor insulation that is not adapted for extreme weather changes; (b) they have limited resources to cope with climate change; and (c) they are more exposed to compromised health from extreme weather patterns due to limited access to health care. For example, climate change can pose a serious health threat through food insecurity and increased toxic air pollution.^11^ Extreme weather can affect crop production resulting in resource scarcity, which can push up costs of food and energy used. In addition, Taylor (2017, https://www.theguardian.com/environment/2017/sep/19/poorest-london-children-face-health-risks-toxic-air-poverty-obesity) highlights that children who live in poverty in the UK are more likely to reside in and attend schools in areas with poor air quality, which can cause development problems in children. These climate change effects further undermine households that are already experiencing poverty in the UK.

Moreover, our results indicate heterogeneous responses of wealth inequality to climate risk shocks between 80–50 (90–50) and 50–20 (50–10) measures in the short term. The results for 80–50 (90–50) ratios show both temperature growth and volatility shocks exert positive and statistically significant effects on wealth inequality immediately after the impact. In contrast, the results for 50–20 (50–10) measures indicate some insignificant or even negative effects of temperature growth and volatility shocks on wealth inequality in a shorter term (e.g., temperature growth shock on 50–10 measures in the 2^nd^ month as reported in Panel A, and temperature volatility shock on 50–10 measures in the 3^rd^ month as reported in Panel B).

As suggested by Islam and Winkel (2017), wealthier households can diversify their assets spacially and financially, and therefore they are less susceptible to damages associated with climate change. Mumtaz and Theophilopoulou (2020) show that in terms of relative size, average net financial wealth and net property wealth are much larger for households towards the right tail of the wealth distribution (i.e., wealthier households), while physical wealth is the largest component of total wealth for households towards the left tail of the wealth distribution. Our results show that, upon the impact of climate shocks, wealth inequality increases significantly between the wealthier households located in the top 20% and 10% percentiles of the wealth distribution relative to the median. The results for 80–50 (90–50) inequality measures could be evidence suggesting that wealthier households located in the top 20% and 10% percentiles of the wealth distribution are less susceptible to damages caused by climate hazards relative to the median, in part because of the diversification of their assets. Upon the impact of climate shocks, wealth inequality between these two groups increases by reducing the wealth of the median households.

**Fig. 3:**
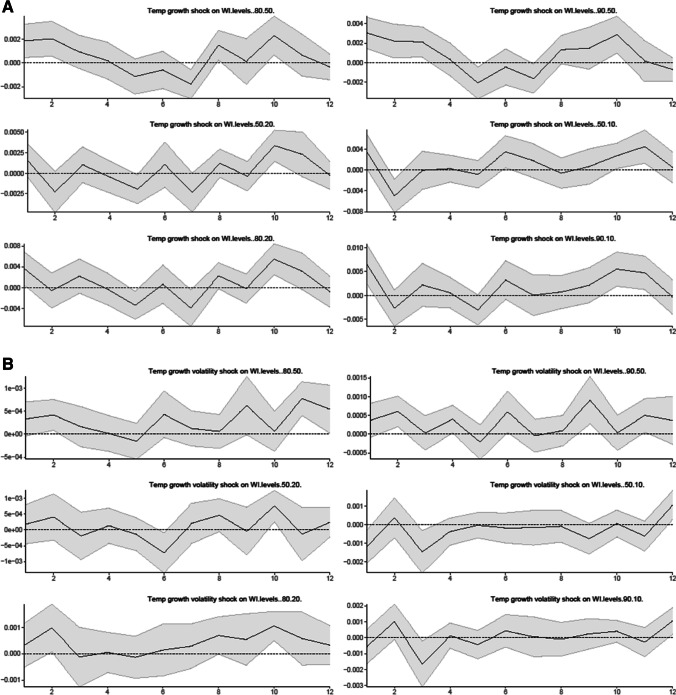
Linear responses of wealth inequality to a climate risk shock (with the control variable).** A** Temperature growth shock on wealth inequality, **B** Temperature growth volatility shock on wealth inequality

To examine if the results reported in Fig. 2 are robust to the influence of the UK macroeconomic and financial shocks, we also include in the model the principal components of 38 economic and financial time series as the control variable. Figure 3 reports the estimated IRFs of wealth inequality to climate risk shocks over 12 months using the models specified in Eq. (2).

The results in Fig. 3 confirm that our results of climate risk shocks on various measures of wealth inequality are robust with the control of industrial production growth in the UK. In the longer term, we find that climate risk shocks increase wealth inequality for all wealth inequality measures. In the short term, the effect of climate risk shocks on wealth inequality is heterogenous across the location of households in the wealth distribution, i.e., climate risk shocks lead to a rise in wealth inequality for wealthier households in the top 20% and 10% percentiles in the right tail of the wealth distribution relative to the median (as captured by the 80–50 and 90–50 ratios), while a shock to climate risk variables can reduce wealth inequality for households near the median relative to the bottom 20% and 10% percentiles in the left tail of the wealth distribution (as captured by the 50–20 and 50–10 ratios).

**Fig. 4:**
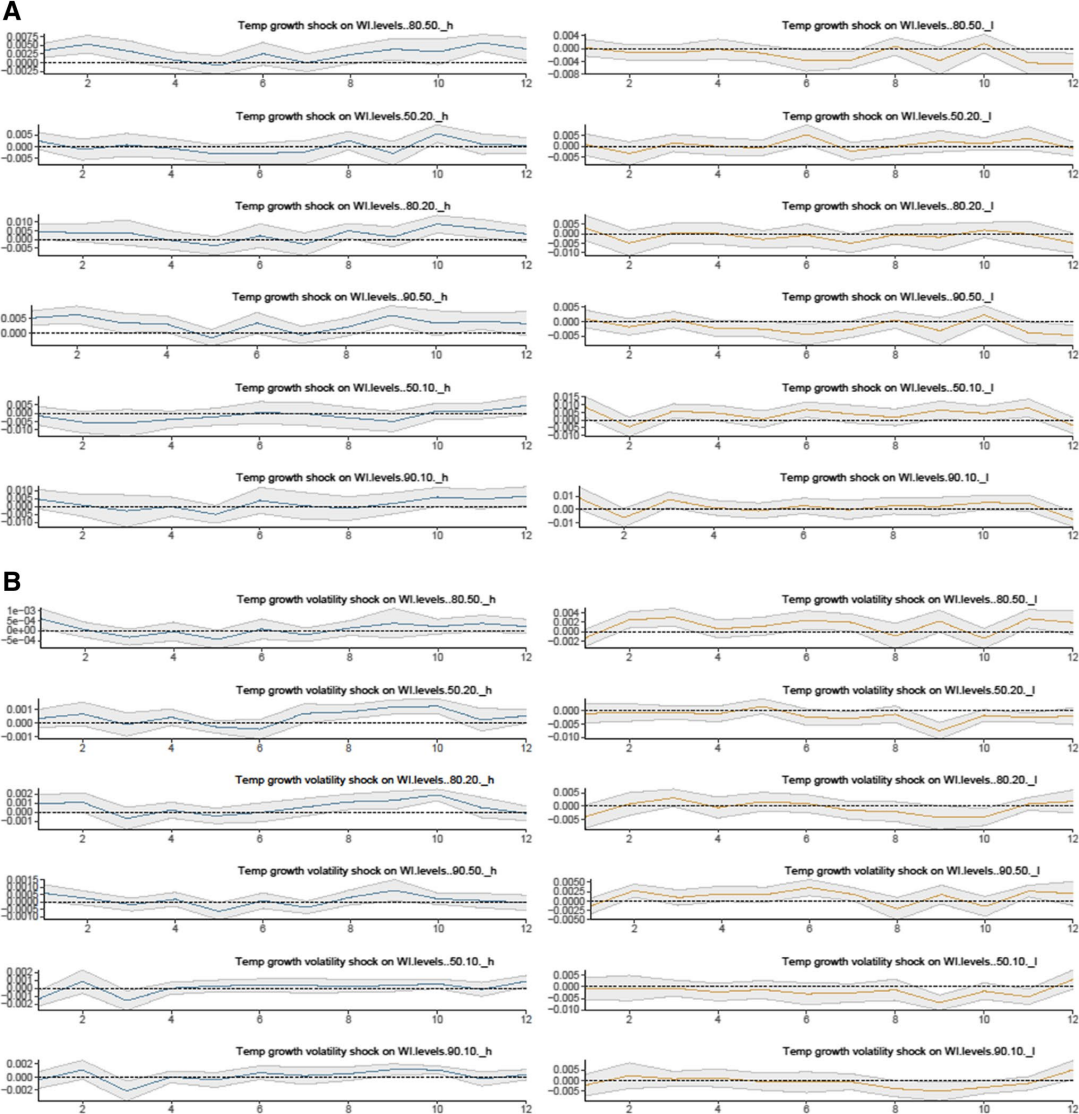
Nonlinear responses of wealth inequality to a climate risk shock (with the control variable). **A** Temperature growth shock on wealth inequality,** B** Temperature growth volatility shock on wealth inequality

Figure 4 depicts nonlinear impulse responses of wealth inequality to a climate risk shock over 12 months by distinguishing the status of climate risk variables in the UK into the high- and low-climate risk regimes.

In Fig. 4, the nonlinear results present some evidence of heterogeneous responses of wealth inequality to climate risk variable shocks between the high- and low-climate risk regimes. The impulse response results show that the positive effects of climate risk shocks on wealth inequality are stronger in the high climate risk regime than in the low climate risk regime. We find that wealth inequality ratios tend to be more sensitive to climate risk shocks in the high climate risk regime compared to the low climate risk regime. Our findings coincide with those of Paglialunga et al. (2020, 2022) who report warmer temperatures (e.g., in the high climate risk regime) have a stronger impact on within-country inequality. Our results also align with the study of Burzyński et al. (2022) who suggest that the consequences of economic and social damages (e.g., inequality) caused by climate change (e.g., temperature changes) are likely to be nonlinear, and can vary over time.

## Additional analysis

**Fig. 5:**
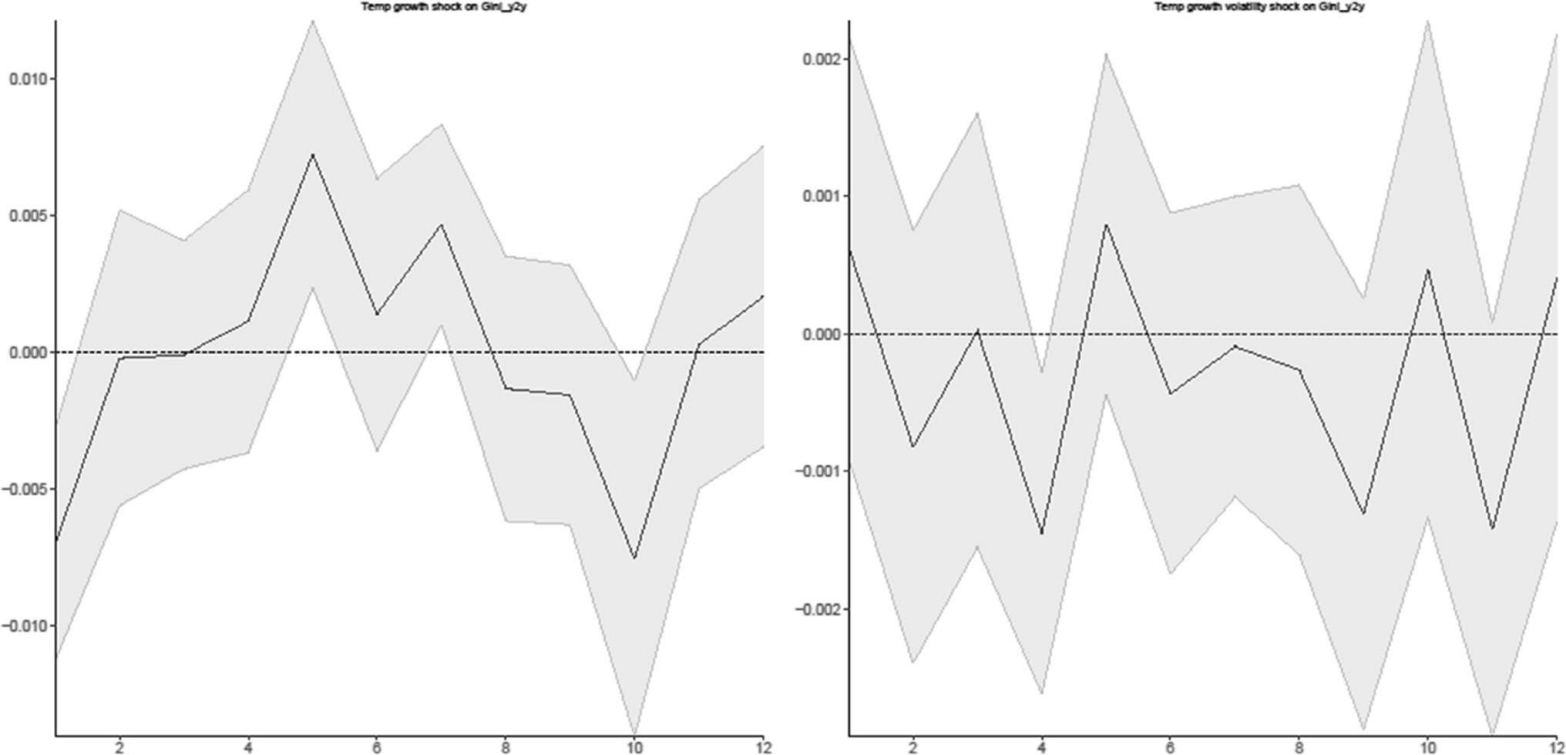
The responses of the wealth Gini to climate risk shocks at a monthly frequency

We also conduct additional analysis using the UK wealth Gini coefficient as an alternative measure of wealth inequality.^12^ In Fig. 5, we report the effects of climate risk shocks on the UK wealth Gini coefficient (as measured by the year-to-year growth) at a monthly frequency. Our results show that the impacts of climate risk shocks on wealth inequality are fluctuating across zero. However, it is noteworthy that the Gini measure is a rather general measure of inequality that does not consider the heterogenous response of inequality across the location of households in the distribution. As pointed out by Mumtaz and Theophilopoulou (2020), the Gini measure does not provide information about the location of households in the wealth distribution that are most affected by inequality. Thus, it is not a preferred measure of wealth inequality compared to the percentile ratios used in our study.

In addition, we employ alternative measures of inequality such as income inequality and consumption inequality to estimate the effects of climate risk shocks on inequality in the UK. We use the quarterly dataset on various measures of UK income inequality and consumption inequality (including the metrics involving the Gini coefficient, the standard deviation of log inequality measures, and the difference between the 90 and 10th percentile of log inequality), as developed by Mumtaz and Theophilopoulou (2017) over the sample period from January 1975 to January 2016.

**Fig. 6:**
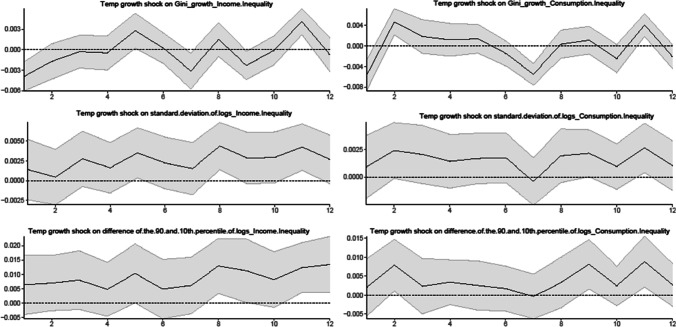
The responses of income inequality and consumption inequality to climate risk shocks at a quarterly frequency

In Fig. 6, we also find qualitatively similar results based on the quarterly dataset of Mumtaz and Theophilopoulou (2017) on various measures of income inequality and consumption inequality. The results show that while using the Gini coefficient measure, the impacts of temperature growth shock on income inequality and consumption inequality display a similar pattern to the impacts of temperature growth shock on wealth inequality (i.e. the effects are fluctuating across zero). Moreover, we observe that the impacts of the temperature growth shock on various measures of income inequality and consumption inequality are all positive and statistically significant in the longer term.

## Conclusion

We examine the effects of temperature shocks on wealth inequality in the UK using high-frequency monthly data. Using the local projections method to compute the IRFs, we observe that both temperature growth and volatility shocks have positive and statistically significant effects on wealth inequality in the long run across the different wealth distributions. However, we also find that the response of wealth inequality to a temperature shock is larger for the poorest households relative to the richest households. In addition, the nonlinear results show that the effects of temperature shocks on wealth inequality are relatively stronger in the high-climate risk regime compared to the low-climate risk regime. These results are robust to the inclusion of the principal components of 38 economic and financial time series as a control variable.

The findings from this study highlight several implications. First, poorer households are more vulnerable to the adverse effects from climate change relative to the rich, because they have limited resources to recover from and adapt to the extreme weather changes. Second, these effects are even more acute for the poor households that are located in high-climate-risk areas, where exposure to climate-related illnesses is more likely, such as heat strokes, or where droughts and floods can affect food security. Third, although wealthier households are also affected by climate change, they are able to diversify their resources, making them less susceptible to climate risk.

As such, a final implication drawn from our findings is that policies need to be adapted to ensure that those with the fewest resources are protected from the risks of climate change. For example, providing housing with better insulation for the poor can cut energy costs related to climate change. Better and affordable health insurance can reduce health inequality exacerbated by climate change. Inclusive access to finance can assist the poorest with the growing cost of food caused by climate change. Moreover, the redistribution of revenues from the carbon tax to the poorest can offset the inequality-aggravating impacts of climate change, while mitigating the risk of rising inequalities within and between countries in the future.

Although this study may not be exhaustive, the findings open up avenues for future research, such as assessing regional differences in temperature and linking these differences to the observed wealth inequality across regions.

## Data Availability

The datasets used and/or analyzed in the current study are all publicly available as indicated in the data segment of the paper, and are also available from the corresponding author on a reasonable request.

## Footnotes

1 https://www.economicsobservatory.com/how-does-climate-change-shape-inequality-poverty-and-economic-opportunity.

2 Inequality can be defined from different perspectives. Inequality is broadly defined as “differences among people in their command over social and economic resources” (Osberg, 2001). In the discipline of economics, economists generally limit their focus to the concept of economic inequality. In this study we use ineqaulity and economic inequality interchangeably. Economic inequality can be further defined according to different dimensions, such as wealth, income, or consumption. For example, wealth inequality is defined as the inequality in “the distribution of wealth across households or individuals at a moment in time”, while income Inequality is defined as “the extent to which income is evenly distributed within a population” (IMF 2022).

3 https://www.lse.ac.uk/granthaminstitute/news/the-heatwave-faced-by-the-uk-and-the-rest-of-western-europe-must-be-viewed-as-an-unnatural-disaster/.

4 The former arises due to rising temperatures, higher sea levels, heavy storms and floods, and wildfires. The latter arise due to a gradual switch-over to a low-carbon economy and comprise risks due to changes in climate policy, the development of disruptive green technologies, and changing consumer preferences (Semieniuk et al. 2021).

5 See: https://www.ons.gov.uk/peoplepopulationandcommunity/personalandhouseholdfinances/incomeandwealth/bulletins/totalwealthingreatbritain/april2016tomarch2018#glossary, for more information about the WAS, including a glossary of terms (such as net property wealth, net financial wealth, private pension and physical wealth), data sources and collection, etc.

6 For calculating IRFs, the LPs technique of Jordà (2005) does not require restrictive assumptions on the specifications and estimations of the unknown true multivariate system itself and thus has a distinct advantage over the traditional Vector Autoregression (VAR) method.

7 See Jordà (2005) for detailed discussions about the LPs method.

8 See the Appendix of Mumtaz and Theophilopoulou (2020) for the list of macroeconomic and financial time series included.

9 The LPs technique utilises the simple OLS regression estimation method and can easily accommodate nonlinear models with flexible specifications, as used to obtain state-dependent IRFs for the high and low regimes of the climate risk variables.

10 We also use the ordinary least squares (OLS) regression method to examine the magnitude of the effects from the independent variables (i.e., temperature growth and volatility shocks) to the dependent variable (i.e., measures of wealth inequality). We observe some positive and statistically significant effects of the temperature growth shock on the 90-50 and 80-50 measures over the sample period (the results are available upon request). It is noteworthy that the OLS regression analysis offers insights into the marginal effects of the independent variables on the dependent variable, while the LPs IRFs employed in this study allow for the dynamic analysis of the effects of climate risk shocks on inequality.

11 https://www.bradford.gov.uk/environment/climate-change/what-is-climate-change-and-how-will-it-affect-the-uk/

12 The montly data of the UK wealth Gini coefficient is available from Mumtaz and Theophilopoulou (2020) and covers a sample period from July 2006 to March 2018.
